# The Advantages of the Zero-COVID-19 Strategy

**DOI:** 10.3390/ijerph19148767

**Published:** 2022-07-19

**Authors:** Zhaohui Su, Ali Cheshmehzangi, Dean McDonnell, Junaid Ahmad, Sabina Šegalo, Yu-Tao Xiang, Claudimar Pereira da Veiga

**Affiliations:** 1 School of Public Health, Southeast University, Nanjing 210009, China; 2 Center on Smart and Connected Health Technologies, Mays Cancer Center, School of Nursing, UT Health San Antonio, San Antonio, TX 78229, USA; 3 Faculty of Science and Engineering, University of Nottingham Ningbo China, Ningbo 315100, China; ali.cheshmehzangi@nottingham.edu.cn; 4 Network for Education and Research on Peace and Sustainability (NERPS), Hiroshima University, Hiroshima 739-8530, Japan; 5 Department of Humanities, South East Technological University, R93 V960 Carlow, Ireland; dean.mcdonnell@setu.ie; 6 Prime Institute of Public Health, Peshawar Medical College, Warsak Road, Peshawar 25160, Pakistan; jahmad@piph.prime.edu.pk; 7 Faculty of Health Studies, University of Sarajevo, 71000 Sarajevo, Bosnia and Herzegovina; sabina.segalo@fzs.unsa.ba; 8 Department of Public Health and Medicinal Administration, Institute of Translational Medicine, Faculty of Health Sciences, Centre for Cognitive and Brain Sciences, Institute of Advanced Studies in Humanities and Social Sciences, University of Macau, Macao SAR, China; 9 Fundação Dom Cabral—FDC, Av. Princesa Diana, 760 Alphaville, Lagoa dos Ingleses, Nova Lima 34018-006, MG, Brazil

**Keywords:** COVID-19, coronavirus, zero-COVID-19 strategy, virus elimination, public health policies

## Abstract

**Introduction**: To curb the COVID-19 pandemic, countries across the globe have adopted either a mitigation or anelimination policy, such as the zero-COVID-19 strategy. However, further research is needed to systematically investigate the advantages of the zero-COVID-19 strategy in the literature. To bridge the research gap, this study examines the zero-COVID-19 strategy in terms of its advantages as a global anti-pandemic framework. **Methods**: A literature review was conducted in PubMed, PsycINFO, and Scopus to locate academic articles that discussed the advantages of the zero-COVID-19 strategy. Braun and Clarke’s thematic analysis approach was adopted to guide the data analysis process. **Results**: The findings of our study show that the advantages of the zero-COVID-19 strategy range from short-term (e.g., limited virus infections, hospitalizations, and deaths), to medium-term (e.g., reduced presence of other infectious diseases), and long-term (e.g., low incidence of long COVID-19). While local residents mainly leverage these advantages, they also impact the global community (e.g., stable global supply of essentials, such as COVID-19 vaccines). **Conclusions**: COVID-19 is catastrophic, yet controllable. Our study examined the advantages of the zero-COVID-19 strategy from a nuanced perspective and discussed how these advantages benefit both the local and the global community in pandemic control and management. Future studies could investigate the shortcomings of the zero-COVID-19 strategy, especially its unintended consequences, such as adverse impacts on vulnerable populations’ mental health, so that society could more efficiently, economically, and empathetically capitalize on the potential of the zero-COVID-19 strategy for the betterment of personal and public health.

## 1. Introduction

COVID-19 was, and still is, catastrophic [1]. In attempting to control the pandemic, two strategies have been widely adopted; the elimination and mitigation approaches [2]. As the virus continutes to mutate, debates about whether the elimination strategy, such as the zero-COVID-19 strategy adopted by countries such as Australia, China, New Zealand, and Singapore, is preferable to the mitigation strategy, such as the anti-pandemic approaches seen in nations such as India, the United States (U.S.), the United Kingdom (U.K.), and Sweden, which continue to gain momentum [3,4,5,6,7]. The elimination approach focuses on rapid containment and maximum prevention actions against the pandemic. In contrast, the mitigation strategy centers on the utilization of relatively lax or relaxed measures to curb virus transmission [8]. In addition to the intensity of anti-pandemic efforts required [9], these two approaches also differ drastically in terms of their pandemic control objectives: the elimination strategy strives to reduce the pandemic’s health consequences to a “negligible” status. This means that limited or no local transmissions, whereas the mitigation method aims to reduce disease consequences to “a locally acceptable level”, meaning that cases, hospitalizations, and deaths align with local communities’ values and interests [10].

Though both pharmaceutical measures (e.g., vaccines, and treatments such as antivirals) and non-pharmaceutical interventions (e.g., masks and physical distancing mandates) can be commonly seen in countries that adopted either the elimination or the mitigation strategy, they are utilized to achieve different pandemic prevention objectives. Although, throughout the pandemic, anti-pandemic measures utilized to achieve the elimination goal are often referred to as the zero-COVID-19 strategies. However, variations can be seen in the specific control measures individual societies have adopted across the pandemic continuum [11]. On the other hand, due to the considerable differences in how “locally acceptable” COVID-19 transmissions and damages are interpreted in the local communities, the mitigation approaches adopted often show stark differences. While no specific strategy can represent the vastly different COVID-19 countermeasures adopted by countries such as the U.S., the U.K., and Sweden, they nonetheless are known for their aim to “live with the virus” or “live with COVID-19”. Noticeably, similar to the notion of “locally acceptable”, no consensus about what “living with COVID-19” entails has yet been reached.

Naturally, differences between the elimination method and the mitigation approach often stoke debates, which often center on: (1) the comparison of the zero-COVID-19 strategy with the “living with COVID-19” philosophy, and (2) the discussion of the role that politics plays in shaping public health policies (i.e., which pandemic strategy to choose) [12]. For instance, nations that aim to promote the “living with COVID-19” philosophy, along with other mitigation approaches, argue that a high COVID-19 vaccination rate is enough for a society to function. In contrast, countries that endorse the zero-COVID-19 strategy often cite evidence that, as seen in countries such as the U.S., the U.K., and Sweden, particularly amid the spread of the Omicron variant and its subvariants, high vaccination rates could not entirely prevent COVID-19 transmission (e.g., breakthrough cases or COVID-19 infections in vaccinated people). Therefore, non-pharmaceutical interventions should also be prioritized in pandemic control and containment [13,14,15,16]. On the other hand, countries that once adopted or continue to adhere to the zero-COVID-19 strategy valued the framework’s ability to optimally reduce avoidable COVID-19 infections, hospitalizations, and deaths in their societies, especially for vulnerable members, such as the elderly, frail, and immunocompromised.

However, while debates and discussions about the zero-COVID-19 strategy and the “living with COVID-19” philosophy continue to gain momentum. In academia and society at large, there is a dearth of research on why countries should choose one anti-pandemic strategy over the other. In other words, even though substantial knowledge has been gained since March 2020, current literature still could not adequately answer questions such as, “What advantages can the zero-COVID-19 strategy offer to the local residents”? and, “Could the global community benefit from individual countries’ zero-COVID-19 approach”? Therefore, to bridge the research gap, this paper investigates the advantages of the zero-COVID-19 strategy. the current study focuses on one key aspect of the zero-COVID-19 strategy, namely its advantages, as opposed to its disadvantages, as well as unintended consequences of the strategy. In the following sections, discussion is given to outline the methods used to answer the research question, as well as the results and overall insights gained from the process.

## 2. Methods

### 2.1. Data Characteristics

A review of the literature review was conducted to answer the research question. The review focused on scholarly articles that discussed the advantages of the zero-COVID-19 strategy. The initial search included articles published between 11 March 2020,when the World Health Organization first characterized COVID-19 as a pandemic [17], and 1 October 2021. To ensure that the up-to-date studies published during the review process were also considered. A follow-up search was conducted on 27 June 2022. The PubMed, PsycINFO, and Scopus databases were adopted for their inclusive and representative collection of medical publications, especially those focused on COVID-19 [18,19,20]. Two search themes were adopted: the strategy (i.e., the zero-COVID-19 strategy or the elimination policy) and the disease context (i.e., COVID-19). Example search terms utilized can be found in Table 1. In addition to societies that adopted the zero-COVID-19 strategy, such as Australia, Hong Kong, New Zealand, Macau, North Korea, Singapore, and Taiwan, China was extensively examined for its consistency in its adherence to the zero-COVID-19 strategy. In addition to the publication date, the following inclusion criteria were also utilized to screen articles: (1) the article must be published in English, (2) the research must be conducted in the context of COVID-19, and (3) the article must discuss the advantages of the zero-COVID-19 strategy as an integrated disease elimination approach, as opposed to singular countermeasures of the strategy (e.g., studies solely focused on lockdowns [21]) or the mere eventuality of reaching zero COVID-19 cases (e.g., [22]).

### 2.2. Data Analysis

Braun and Clarke’s thematic analysis approach was adopted to guide the data analysis process [23], which includes six steps: (1) become familiar with the data, (2) generate initial codes, (3) identify preliminary themes, (4) review emergent themes, (5) define and name themes, and (6) produce the report. After reviewing the title, abstract, and the full text of the records, we removed articles that did not meet the selection criteria. The remaining articles were subsequently read and examined, focusing on content that discusses the advantages of the zero-COVID-19 strategy. After identifying all relevant sections closely related to the aim of the study, we then applied Braun and Clarke’s analytical approach to identify common themes across the included articles.

## 3. Results

A total of fifty articles were included in the final review, as listed in Table 2. The advantages of the zero-COVID-19 strategy are broadly understood as short-term (e.g., reduced risks of COVID-19 infections, hospitalizations, and deaths), medium-term (e.g., low presence of other infectious diseases), and long-term benefits to society (e.g., the limited presence of COVID-19). A key advantage of the zero-COVID-19 strategy centers on its ability to prevent avoidable virus infections, hospitalizations, and deaths. In a 2022 article, Burki noted that, “China’s policy has been enormously successful. Throughout theentire course of the pandemic, the country of 1.4 billion people has reported 1,655,477 cases of COVID-19 and 13,524 deaths… The nation’s economy grew by a healthy 8.1% last year” [24]. In a modelling study, Cai and colleagues further found that, without zero-COVID-19 measures, such as lockdowns, the city of Shanghai would have to face “a projected intensive care unit peak demand of 15.6 times the existing capacity and causing approximately 1.55 million deaths” amid the Omicron outbreaks [25].

One of the most noticeable medium-term health benefits of the zero-COVID-19 strategy is the reduced presence of infectious diseases (e.g., [26]). In a study of Kawasaki disease, a communicable disease that can cause high fever and coronary vasculitis in children, researchers found that compared to 2018 and 2019, the incidence of the disease in Taiwan decreased by 30% and 31% in 2020, respectively [27]. In terms of long-term benefits, in addition to limiting the presence of long COVID-19 (e.g., [28]), economic considerations are also discussed throughout the literature. In an analysis of 44 countries’ COVID-19 policies, König and Winkler concluded that “countries successfully applying the elimination strategy achieved better health outcomes than their peers without having to accept lower growth” [29].

More targeted comparisons are also studied to offer nuanced insights into the positive impacts of the zero-COVID-19 strategy. In an analysis conducted in New Zealand, for instance, Wilson and colleagues noted that the country’s elimination strategy reached desired success “in both health and economic terms compared to other OECD countries” [30]. While the advantages of the zero-COVID-19 strategy are mainly experienced by local residents, such as vulnerable populations such as the elderly and the immunocompromised, they also impact the global community. Ding and Zhang discuss how China’s zero-COVID-19 strategy has helped slow the global spread of the virus and vaccine production and, by extension, vaccine equity [31].

**Table 2:** The list of articles included in the final review.

Author	Year	Title
AlTakarli [32]	2020	China’s response to the COVID-19 outbreak: a model for epidemic preparedness and management
Anderson et al. [33]	2020	How will country-based mitigation measures influence the course of the COVID-19 epidemic?
Baker et al. [34]	2021	Elimination could be the optimal response strategy for COVID-19 and other emerging pandemic diseases
Burki [24]	2022	Dynamic zero COVID policy in the fight against COVID
Cai et al. [25]	2022	Modeling transmission of SARS-CoV-2 Omicron in China
Cai et al. [35]	2022	China’s ‘dynamic zero COVID-19 strategy’ will face greater challenges in the future
Carlton et al. [36]	2021	Charting elimination in the pandemic: a SARS-CoV-2 serosurvey of blood donors in New Zealand
Chen et al. [37]	2021	Comparison of public health containment measures of COVID-19 in China and India
Chen et al. [38]	2021	A cross-country core strategy comparison in China, Japan, Singapore and South Korea during the early COVID-19 pandemic
Chen et al. [39]	2021	Policy disparities in response to COVID-19 between China and South Korea
Chen et al. [40]	2021	The heterogeneity of the COVID-19 pandemic and national responses: an explanatory mixed-methods study
Chen et al. [41]	2022	China can prepare to end its zero-COVID policy
Cheng et al. [42]	2022	Rapid spread of SARS-CoV-2 Omicron subvariant BA.2 in a single-source community outbreak
Cheshmehzangi et al. [43]	2022	Commentary: China’s zero-COVID approach depends on Shanghai’s outbreak control
Das [44]	2022	COVID-19 and the elderlies: how safe are Hong Kong’s care homes?
Ding et al. [31]	2022	China’s COVID-19 control strategy and its impact on the global pandemic
Dyer [45]	2022	COVID-19: Lockdowns spread in China as omicron tests “zero COVID” strategy
Fitzgerald et al. [46]	2020	COVID-19: A tale of two pandemics across the Asia Pacific region
Hale et al. [47]	2021	A global panel database of pandemic policies (Oxford COVID-19 Government Response Tracker)
Hassan et al. [48]	2021	Hindsight is 2020? Lessons in global health governance one year into the pandemic
Islam et al. [9]	2020	Variations in COVID strategies: Determinants and lessons
Jecker et al. [49]	2022	Does Zero-COVID neglect health disparities?
König et al. [29]	2022	The impact of government responses to the COVID-19 pandemic on GDP growth: does strategy matter?
Lee et al. [50]	2020	Should countries aim for elimination in the COVID-19 pandemic?
Lu et al. [51]	2021	COVID-19 in Germany and China: mitigation versus elimination strategy
Mallapaty [52]	2022	China’s zero-COVID strategy: what happens next?
Mason et al. [26]	2022	Reduced presentations with fractures or orthopaedic infections to a major children’s hospital during a national COVID-19 elimination strategy
McKee [28]	2020	Achieving zero COVID is not easy, but the alternative is far worse
Müller et al. [53]	2020	COVID-19 control: can Germany learn from China?
Normile [54]	2021	‘Zero COVID’ is getting harder—but China is sticking with it
Normile [55]	2022	Can ‘zero COVID’ countries continue to keep the virus at bay once they reopen?
Normile [56]	2022	China quietly plans a pivot from ‘zero COVID’
Schaefer [57]	2022	Zero COVID and health inequities: lessons from Singapore
Shimizu et al. [58]	2021	Japan should aim to eliminate COVID-19
Shokoohi et al. [59]	2020	COVID-19 pandemic: what can the West learn from the East?
Stobart et al. [60]	2022	Australia’s Response to COVID-19
Taylor [61]	2022	COVID-19: Hong Kong reports world’s highest death rate as zero COVID strategy fails
Wan et al. [62]	2022	Diagnostic strategy of SARS-CoV-2 for containment under China’s zero-COVID-19 policy
Wang et al. [63]	2020	Policy disparities in fighting COVID-19 among Japan, Italy, Singapore and China
Wong et al. [64]	2022	Transmission of Omicron (B.1.1.529)—SARS-CoV-2 Variant of Concern in a designated quarantine hotel for travelers: a challenge of elimination strategy of COVID-19
Yang et al. [27]	2021	Public health interventions for COVID-19 reduce Kawasaki disease in Taiwan
Yuan [61]	2022	Zero COVID in China: what next?
Zhan et al. [65]	2022	Zero-COVID strategy: what’s next?
Zhang et al. [66]	2021	Policy disparities in response to the first wave of COVID-19 between China and Germany
Zhang et al. [67]	2021	Policy disparities in response to the first wave of COVID-19 between China and Germany
Zhang et al. [68]	2022	Asymptomatic transmissibility calls for implementing a Zero-COVID strategy to end the current global crisis

## 4. Discussion

In this study, we set out to examine the advantages of the zero-COVID-19 strategy. While the strategy has advantages and disadvantages, to ensure research focus, we closely focused on the potential of the zero-COVID-19 strategy to help society better cope with the pandemic. To our knowledge, this is one of the first studies discussing the benefits of the zero-COVID-19 strategy from a nuanced perspective. In the following sections, we will discuss the research findings in greater detail, with a close focus on the advantages of the zero-COVID-19 strategy from the lens of: (1) short-term, (2) medium-term, and (3) long-term benefits to the local and the global community.

### 4.1. Short-Term Benefits

One of the most pronounced short-term advantages of the zero-COVID-19 strategy is its ability to prevent avoidable virus infections, hospitalizations, and deaths (see Table 3). Overall, compared to individuals living in societies that adopt a “living with COVID-19” mitigation policy (e.g., the U.S.), individuals often have a considerably lower presence of COVID-19 when the zero-COVID-19 strategy is applied [69,70,71]. For instance, in an analysis of COVID-19 data from 72 countries that were accumulated in 2020, researchers found that, compared to other anti-pandemic measures, the elimination strategy offers substantially greater protection for members of the society, evidenced by indicators ranging from COVID-19 deaths, income, unemployment, trust, as well as mental and physical health at the population level [69]. In general, public health advantages associated with the zero-COVID-19 strategy mirror the essence and the namesake of the umbrella term: the elimination policy aims to eliminate the pandemic [9], including COVID-19 infections, hospitalizations, and deaths. The mitigation policy, on the other hand, sets out to mitigate the pandemic [9]; by not aiming to interrupt the virus transmission patterns (i.e., the elimination of COVID-19 cases), this approach essentially assigns normalcy to COVID-19 infections, as seen in the “Freedom Day” sponsored by the U.K. government [72].

### 4.2. Medium-Term Benefits

It is also important to note that hikes in COVID-19 cases, hospitalizations, and deaths often translate into heightened demand for medical services, which could result in substantial burdens on COVID-19 patients, non-COVID patients, and health care professionals [73,74,75]. Take health professionals such as doctors and nurses, for instance. Recurring evidence shows that health care professionals have to shoulder substantially more physical and psychological stress when COVID-19 infection, hospitalization, and death rates rise [76,77,78]. In a comparison study on breast cancer patients, COVID-19 frontline nurses, and non-frontline nurses, results show that both the patients and the COVID-19 frontline nurses have experienced greater levels of psychological issues (e.g., depression) compared with non-frontline nurses [79]. Evidence further indicates that, compared to non-medical workers, health professionals often experience greater adverse health outcomes, ranging from higher rates of anxiety and depression to insomnia [80,81,82,83].

In addition to the workload associated with COVID-19, mounting evidence shows that in countries that utilized the mitigation strategy (e.g., the U.S.), health care professionals often have to make agonizing, if not impossible, decisions–such as rationing the oxygen supply or intensive care unit beds—essentially being forced to determine who should live and who should die [84]. However, in light of the unintended consequences that the zero-COVID-19 strategy could cause, such as a high demand for health care professionals for testing and tracing, more rigorous empirical evidence is needed to reach a more grounded conclusion in terms of whether the strategy also has a positive impact on the physical and psychological health of health professionals and other vulnerable populations, including COVID and non-COVID patients. Other than potentially reduced knock-on effects on the overall health and social infrastructure, medium-term advantages of the zero-COVID-19 strategy, such as a reduced presence of other infectious diseases and relatively undisturbed economic productivity, have also been discussed in the literature [26,27]. In addition to the reduced presence of health conditions, such as fractures and Kawasaki disease [26,27], it is possible that the relatively low presence of monkeypox in zero-COVID-19 countries such as China may also be contributed to the zero-COVID-19 strategy’s rigorous capability in curbing the transmission of viruses.

### 4.3. Long-Term Benefits

While a wide array of achievements could be contributed to the zero-COVID-19 policy, the continuity of economic activity is one of the most noticeable advantages of the zero-COVID-19 strategy from a long-term perspective. Across the pandemic, countries such as the U.S. and the U.K. have faced severe bouts of disruptions to their medical infrastructure, ranging from shortages of face masks, depleted oxygen supplies, and overpacked intensive care units, to decade-long backlogged non-COVID-19-related surgeries [85,86,87]. By contrast, these shortages are considerably less likely in zero-COVID-19 strategy countries. Take China for instance. Rather than debilitated by shortages of critical medical goods and supplies, China emerged as one of the countries that not only produced, but also donated a wide range of medical essentials, such as COVID-19 vaccines [88]. Another long-term benefit of the strategy that is relatively poorly studied is the low presence of long COVID-19 in zero-COVID-19 strategy countries such as China.

While our understanding of the pandemic is still unfolding, recurring evidence shows that long COVID-19 is extremely prevalent among infected populations [89,90,91]. Long COVID-19 refers to a wide range of sustained symptoms, ranging from fatigue, dyspnea, joint pain, chest pain, and anosmia, to cognitive impairment, that “occurs in individuals with a history of probable or confirmed SARS-CoV-2 infection, usually 3 months from the onset of COVID-19 with symptoms and that last for at least 2 months and cannot be explained by an alternative diagnosis” [92]. In a study of approximately 2 million people in the U.S., for instance, researchers found that among participants who had contracted COVID-19, approximately 1 in 5 of those aged 18–64 years and 1 in 4, aged 65 years and over, experience at least one long COVID-19 symptom [93]. This means that, while some mitigation countries, such as Sweden, might have a relatively less daunting toll of COVID-19, especially when compared to nations such as the U.S. (see Table 3), they may still have to face long-term issues, such as long COVID-19. Moreover, factoring in current debates on whether to classify long COVID-19 as a form of disability, it is safe to conclude that one of the long-term advantages that the zero-COVID-19 strategy provides to society is the considerably limited presence of long COVID-19.

### 4.4. Concluding Thoughts

The zero-COVID-19 strategy holds great potential and promise, as discussed in our study. Essentially, the strategy’s ability to effectively curb virus spread, and in turn, prevent avoidable COVID-19 morbidity and mortality rates, not only explains why it has been adopted widely amid the current pandemic, but also shows its applicability for future infectious disease outbreaks. The relevance of elimination methods, such as the zero-COVID-19 strategy, could be particularly pronounced amid the early days of outbreaks, when knowledge about the disease is still accruing, testing and tracing capabilities are still building, and effective pharmaceutical interventions, such as vaccines, are either absent, or still under development. As seen amid the worldwide monkeypox outbreaks in 2022, for instance, due to poor understanding of what might have caused the erratic behaviors of the virus [94], even countries that are known for their “live with COVID-19” philosophy, such as the U.S. and the U.K., have advised strict non-pharmaceutical measures, such as self-quarantine, to help at-risk populations better navigate their infections [95]. This, in turn, further suggests that, rather than a privileged pandemic strategy that is only compatible with certain societies, the zero-COVID-19 strategy can be readily applied to most, if not all, societies. From a theory-building perspective, these insights could further enrich the classification and application of pandemic control methods, which are often categorized in terms of templated guidelines (e.g., mitigation vs. elimination policies), as opposed to flexible toolboxes.

It is also important to note that the adoption of the zero-COVID-19 strategy is not a guarantee for sustained pandemic control success. Many factors could shape the formation and impacts of pandemic control policies [58,96,97,98]. For instance, in addition to the makeup and structure of anti-pandemic measures, differences in policy administration and prioritization of public health may also explain why there are variations in zero-COVID-19 countries’ pandemic performances [76]. We also noticed that, in discussing the advantages of the zero-COVID-19 policy, articles often either explicitly or inexplicitly assumed government and health officials’ respect for and prioritization of public health as a pretext for adopting or adhering to the zero-COVID-19 strategy. Whether this assumption mirrors reality requires trustworthy evidence to prove, which could serve as a direction for future research. Overall, it is our hope that insights provided in our study, as well as those from future research, could help society more confidently dissect questions such as whether nations in the Middle East (e.g., South Arabia), Far East (e.g., Japan), South Asia (e.g., Thailand), Sub-Saharan Africa (e.g., Sudan), and Europe in general (e.g., Sweden) could also benefit from the zero-COVID-19 strategy, or the elimination policy in general, during COVID-19 or future pandemics.

### 4.5. Limitations

While our study bridges important gaps in the literature, it is not without limitations. First, the findings of this study are solely focused on the advantages of the zero-COVID-19 strategy. It is important to note that the policy has many downsides as well, as discussed in the literature [77,78], such as its disruptions to people’s routines and business practices as a result of rigorous adherence to physical distance mandates (e.g., high Stringency Index scores [99]). In addition to unintended consequences, such as loss of access to essential health and social services, mental health challenges caused by strict quarantines and lockdowns, especially poorly executed ones seen in Shanghai amid the city’s Omicron outbreaks [100], should also be extensively investigated to prevent future (preventable) miseries across social sectors. However, as the downsides of the zero-COVID-19 strategy are not within the scope of our research question, they were not investigated in the current study. Last but not least, in light of the uncertainties associated with COVID-19 (e.g., breakthrough cases [101]), it is possible that novel advantages and/or disadvantages of the zero-COVID-19 strategy may emerge as the pandemic evolves. Researchers could update the impact of the policy for society at large in a timely fashion to address this issue.

## 5. Conclusions

COVID-19 is catastrophic, yet the adverse impacts of the pandemic are both containable and controllable. Our study examined the advantages of the zero-COVID-19 strategy from a nuanced perspective, and discussed how these advantages benefit both the local and the global community in pandemic control and management. To gain a more balanced understanding, future studies could investigate the shortcomings of the strategy, especially its unintended consequences, such as adverse impacts on public mental health, so that society could more efficiently, economically, and empathetically capitalize on the zero-COVID-19 strategy for the betterment of personal and public health in the long run.

## Figures and Tables

**Table 1:** Example search terms adopted.

Theme	Search String
Zero-COVID- strategy	“zero-tolerance” OR “zero tolerance” OR “zero-COVID” OR “zero COVID” OR “zero COVID-19” OR “zero-coronavirus” OR “zero coronavirus” OR “elimination policy” OR “elimination strategy”
COVID-19	“COVID-19” OR “novel coronavirus 2019” OR “coronavirus 2019” OR “SARS-CoV-2”

**Table 3:** COVID-19 infections and death data as of 25 June 2022.

Country	Total Confirmed Cases	Total Confirmed Cases per Million People	Cumulative Confirmed Deaths	Cumulative Confirmed Deaths per Million People	Share of People Vaccinated against COVID-19 *
China	888,120	614.95	5226	3.62	90%
Sweden	2.52 million	247,611.18	19,075	1879.20	77%
U.S.	86.95 million	261,174.98	1.01 million	3051.63	78%
U.K.	22.67 million	332,387.03	179,961	2640.56	79%

* Vaccination data as of 23 June 2022; data: Our World in Data, Oxford University.

## Data Availability

Data are available upon reasonable request.

## Abbreviations

U.S.	The United States
U.K.	The United Kingdom
